# Phenotypic Switching Resulting From Developmental Plasticity: Fixed or Reversible?

**DOI:** 10.3389/fphys.2019.01634

**Published:** 2020-01-22

**Authors:** Warren W. Burggren

**Affiliations:** Developmental Integrative Biology, Department of Biological Sciences, University of North Texas, Denton, TX, United States

**Keywords:** development, plasticity, evolution, environment, critical window

## Abstract

The prevalent view of developmental phenotypic switching holds that phenotype modifications occurring during critical windows of development are “irreversible” – that is, once produced by environmental perturbation, the consequent juvenile and/or adult phenotypes are indelibly modified. Certainly, many such changes appear to be non-reversible later in life. Yet, whether animals with switched phenotypes during early development are unable to return to a normal range of adult phenotypes, or whether they do not experience the specific environmental conditions necessary for them to switch back to the normal range of adult phenotypes, remains an open question. Moreover, developmental critical windows are typically brief, early periods punctuating a much longer period of overall development. This leaves open additional developmental time for reversal (correction) of a switched phenotype resulting from an adverse environment early in development. Such reversal could occur from right after the critical window “closes,” all the way into adulthood. In fact, examples abound of the capacity to return to normal adult phenotypes following phenotypic changes enabled by earlier developmental plasticity. Such examples include cold tolerance in the fruit fly, developmental switching of mouth formation in a nematode, organization of the spinal cord of larval zebrafish, camouflage pigmentation formation in larval newts, respiratory chemosensitivity in frogs, temperature-metabolism relations in turtles, development of vascular smooth muscle and kidney tissue in mammals, hatching/birth weight in numerous vertebrates,. More extreme cases of actual reversal (not just correction) occur in invertebrates (e.g., hydrozoans, barnacles) that actually ‘backtrack’ along normal developmental trajectories from adults back to earlier developmental stages. While developmental phenotypic switching is often viewed as a permanent deviation from the normal range of developmental plans, the concept of developmental phenotypic switching should be expanded to include sufficient plasticity allowing subsequent correction resulting in the normal adult phenotype.

## Development, Plasticity and Phenotype

That developing animals are shaped by their environment is not a new concept. Aristotle deserves first credit for recognizing that animals will develop different forms when raised in different environments (Aristotle, 350BC). Of course we now frame these observations as changes in gene expression influenced by environmental stressors. In this essay I first briefly expand upon the Gene X Environment context to include development, epigenetics and other factors, and then consider whether the product of these interactions (e.g., modified phenotypes enabled by developmental plasticity) are fixed or reversible.

### Moving Beyond the Gene–Environment Interaction Paradigm in the Context of Development

Phenotypic plasticity is a venerable, long-standing concept, and is often framed as the interactions of genes (G) by environment (E), creating a specific phenotype that is the result of so-called ‘G × E’ interactions. This concept has been reviewed dozens of times as both a general concept as well as in specific contexts such as human pathologies and disease – an entry into the voluminous literature can be gained from Moffitt et al. (2005), Manuck and McCaffery (2014), Halldorsdottir and Binder (2017), Josephs (2018), and Saltz et al. (2018). Much of the discussion of environmentally induced changes in gene expression resulting in modified phenotype does not explicitly involve a developmental component, though certainly much is known about developmental phenotypic plasticity in both plants and animals – for reviews see West-Eberhard (2005), Spicer and Rundle (2007), Uller (2008), de Jong and Leyser (2012), Bateson et al. (2014), Standen et al. (2014), Burggren and Mueller (2015), Bateson (2017), and Burggren (2018). Thus, for developing animals, the classic G × E framework is expanded to the more encompassing (Genes × Environment) × Development, or (G × E) × D. Note that it has been suggested that Time (T) and even allometry be a component of this construct (Pigliucci et al., 1996), but in this essay we consider development as a distinct sub-set of time (Dubansky, 2018).

It is becoming increasingly clear that, to this basic formula describing developmental phenotype, we must add an *epigenetic* component (‘Epi’) representing intragenerational and especially intergenerational environmental experiences resulting in modified gene expression (Skinner, 2011; Jablonka, 2013; Burggren and Crews, 2014; Skinner, 2015; Burggren, 2016, 2017). The influences of epigenetic readers, writers and erasers on the epigenetic markers that influence gene expression can result from changes ranging from minutes to across multiple generations. Thus, the formula G × E × D, which assumes that gene expression results from contemporaneous environmental conditions, can be expanded to G × (E + Epi) × D. This new expression now includes both within-life altered gene expression patterns as well as both known and unknown epigenetic factors from past parental/ancestral experiences through altered gene expression patterns in germ cells. These epigenetic changes, combined with ambient environmental affects on gene expression, contribute to the control of the phenotype that emerges during development. Importantly, these genomic/epigenomic interactions form the basis for the differing phenotypic outcomes that lead to successful survival in stressful environments.

Finally, an additional source of phenotype switching has been proposed to emerge from stochastically driven cell differentiation during early development that is amplified as tissue differentiation and growth progresses (Woods, 2014). Developing animals might then be a mosaic of both programed and stochastic development. As a consequence, the originally lauded but now limiting ‘G × E’ construct could be expanded to G × (E + Epi) × (D × S), where S represents the influence of stochasticity. In suggesting this, it is realized that there will doubtlessly be disagreement as to where the brackets are placed in this ‘formula’, whether the × symbols should in fact be + signs, etc. etc. The point of this or any other next-generation formulae encompassing these concepts is that the G × E paradigm is now more properly regarded as an overly simplistic, historical view of how animals interact with their environment during development and maturation.

All of these factors – genes, environment, development, epigentic markers and stochastic changes in the developmental plan – interact together in complex and sometimes unpredictable ways, and lead to modified phenotypes resulting from developmental phenotypic plasticity. Importantly, however, there is also a crucial element of *timing*, in that the expression of genes, appearance of stressors in the environment, progression of development, and the writing and erasing of epigenetic markers all march to the beat of a different drummer. No where is this more evident than in the concept of “critical windows” during development, which will now be explored.

### Critical Windows, Developmental Plasticity, and Phenotype Switching

To heavily paraphrase T. Dobzhansky’s famous phrase on biology and evolution (Dobzhansky, 1973), nothing in *development* makes sense except in the light of *critical windows* (also known as sensitive periods). The terms ‘critical moment’ and ‘sensitive period’ were first used in the early 20th century by the influential embryologist Charles Stockard, who showed that oxygen deprivation during certain specific periods of development created developmental anomalies in *Fundulus*, trout and frogs (Stockard, 1921). These observations helped explain the relationship between time in development and susceptibility to environmental influences, a relationship further explored in Nobel laureate Hans Spemann’s *Embryonic Development and Induction* (Spemann, 1938). Likely drawing upon the analogy with biology, in the late 1950s neurologists Wilder Penfield and Lamar Roberts advanced the “critical period hypothesis” related to neural development and language acquisition (Penfield and Roberts, 1959). Lenneberg (1967) further promoted this notion for human development in his book *Biological Foundations of Language*. Noteworthy is that, somewhat in contrast to the embryological literature that focuses on maladaptations (usually morphological) emerging during critical windows, in the human linguistics and psychological literature, the critical window is often regarded as a period when certain environmental exposures are actually necessary and important for language development and other aspects of normal development, e.g., Hensch and Bilimoria (2012). Currently, investigators typically describe a critical window as a specific, defined period during development when G × E × D (and, studied less often, [G × (E + Epi) × D] interactions result in subsequently switched (modified) phenotypes that differ from the normally expected range of developmental trajectory. Numerous authors have offered up similar definitions – e.g., Kunes and Zicha (2006), Ferner and Mortola (2009), Ali et al. (2011), Burggren and Reyna (2011), Vickers (2011), Voss (2013), Alvine and Burggren (2014), Burggren et al. (2014), Burggren and Mueller (2015), Eme et al. (2015), and Pelster and Burggren (2018).

The concept of critical window is the subject of considerable investigation in disciplines ranging from physiology to ecology to toxicology. Indeed, at the time of the writing of this article, the Pubmed data base listed >4400 articles evoking critical windows (the majority being in animal rather than plant development), and >32,000 articles describing sensitive periods in a human psychological context. While there is huge variation in observations and experiments on critical windows and their effects, Table 1 outlines some of the key general characteristics of critical windows, along with some examples of resulting phenotype switching.

**TABLE 1:** Characteristics of critical windows for development.

**Characteristics of critical windows**	**Description**	**Examples**	**References**
Timing in Development of Critical Windows	Critical developmental windows can occur from shortly after egg fertilization until achievement of sexual maturity.	Human psychiatric illnesses frequently first manifest during critical windows in teenagers, potentially associated with gut microbiome dysfunction.	McVey Neufeld et al., 2016
		Human cardiovascular form and function impacted by environmental toxicants during critical windows starting as early as week 2 after conception	Lage et al., 2012

Duration (‘Width’) of Critical Window	Finite “width” to critical window – i.e., distinct onset and closing of window, but interpretation of critical window “edges” is dependent upon stressor dose	Cardiac development in chicken embryos primarily sensitive during week 2 of 3 weeks incubation	Chan and Burggren, 2005
		Gonad differentiation in zebrafish between 30–44 days post-fertilization	Quintaneiro et al., 2019
		Modeling of critical window as a 3D construct of time, dose and phenotype	Burggren and Mueller, 2015

Duration of Switched Phenotype	Phenotypic switching irreversible, persisting through subsequent life stages	Larval hypoxia has long-term effects on protein digestion and growth in juvenile European sea bass	Zambonino-Infante et al., 2017
		Chicken embryos show aberrant aortic arch morphogenesis when hemodynamic variables are manipulated specifically at Stage 21.	Kowalski et al., 2013

Number of Critical Windows Per Trait	Typically only one, but multiple critical windows can exist for same trait	Lipid and glucose metabolism in adult sheep is similarly affected by undernutrition early in gestation as well as immediately postnatally	Poore et al., 2010
		Correction of structural abnormalities in mouse brain cortex have multiple critical windows	Cox et al., 2018

Stressors Acting During Critical Window	Stressors can be intrinsic or extrinisic (environmental) factors.	Odors (aversive or attractive) in first week post-eclosion fruit fly larvae alter olfactory circuitry	Golovin and Broadie, 2016
		Hypoxia during middle third of avian incubation alters gross morphology and metabolic physiology	Dzialowski et al., 2002

Dose Effects During Critical Window	Phenotypic switching during critical windows is dose-dependent	Body mass changes in *Artemia* during early development are dependent on strength of environmental salinity	Mueller et al., 2016
		Hypoxia-induced alteractions of morphology and physiology of chicken embryo show differential responses to 13 and 15% oxygen	Zhang and Burggren, 2012

Sex Differences in Critical Window Susceptibility for Same Trait	Phenotypic switching during critical windows is sex-dependent	Prenatal critical window for oranotin toxicant exposure in rats results in greater permanent phenotype switching in males compared to females	Grote et al., 2009
		Prenatal critical window for particulate air pollution exposure causes phenotype switching in human male but not female children	Hsu et al., 2015

Organ System Differences in Critical Windows	Timing of development of window differs between organ systems within an organism	Critical window for hypoxic effects on heart mass and blood pressure are considerably different in timing and width in embryonic alligator hearts	Tate et al., 2015
		Critical windows for sensitivity to environmental toxicant differ in timing and duration for immune and respiratory systems in humans	Dietert et al., 2000

Population Differences in Critical Windows For Same Trait	Timing and width of critical window for a particular phenotypic trait varies between different populations – i.e., “heterokairy”	Human populations differ in critical window for infant weight gain and its effect on adult adiposity	Wells, 2014
		Hypersalinity delays onset of heartbeat and changes timing of foot attachment and eye spot formation in the euryhaline snail *Radix balthica*	Tills et al., 2010

Species Differences in Critical Windows For Same Trait	Timing of window for a particular phenotypic trait varies between different species – i.e., “heterochrony”	Critical windows for nephrogenesis and morphologica renal development differ between dog, pig, rabbit, monkey, mouse, and rat	Frazier, 2017
		Critical windows for motor activity and motor function performance identified by exposure to environmental neurotoxins differ in rats and mice	Ingber and Pohl, 2016
		Critical window for gut microbiome establishment differs between wood frogs, green frogs and bullfrogs	(Ingber and Pohl, 2016)

## Are Changes That Are Evoked During Critical Windows Reversible or Fixed?

### Definitions and Semantics

Most organisms typically have at least some capacity to counteract potentially negative effects of environmental fluctuations that they experienced during development or even later in life as adults. Yet, a commonly (though not universally) posited key characteristic of developmental phenotypic plasticity is that a switched phenotype produced specifically as a result of developmental plasticity is essentially *irreversible* – e.g., Wilson and Franklin (2002); West-Eberhard (2003), Matesanz et al. (2010, 2012), Utz et al. (2014), Woods (2014), Senner et al. (2015), Beaman et al. (2016), Slotsbo et al. (2016), and Noh et al. (2017). This view is especially held to be true when prevalent when phenotypic switching occurs during a narrow developmental window. Interestingly, Woods (2014) employs the term phenotypic *flexibility* for reversible (or additional new) phenotypic changes. Utz et al. (2014) uses the term *reversible* if a trait’s phenotype “…*can be reversed to the original state.*” Some authors do not distinguish between ‘plasticity’ and ‘flexibility’ or even see the need to, whereas others painstakingly lay out their ground for the irreversibility of phenotypic modification during development – for discussion, see Debat and David (2001), Gabriel (2005), Woods (2014), and Sommer et al. (2017). Investigators studying cold hardening in developing insects have differentiated between irreversible “*developmental acclimation*” and reversible “*short-term acclimation*” (Noh et al., 2017). Finally, several studies have differentiated between ‘developmental’ and ‘reversible’ environmentally induced phenotypic modification (Angiletta, 2009; Munday et al., 2013; Utz et al., 2014; Donelson et al., 2017).

Regarding definitions, it is not the intention of this review to add to the existing definitional morass surrounding the definition of developmental phenotypic plasticity and resultant phenotypic switching^1^ (Korzybski, 1933). Yet, perhaps the additional term ‘correctable’ should be inserted into the discussion of terms. Why? Correctable indicates that the normal range of adult phenotypes could still be achieved despite potentially transient phenotypic modification during development – that is, the switched phenotype can be corrected to the phenotype that would result if gene expression had not been modified by environmental or epigenetic influences. This would leave the term ‘reversible’ to apply to the unusual phenomenon of a true reversal (backtracking) along a developmental pathway (which can indeed occur, as discussed below). Yet, the problem with this reversible/correctable semantic conundrum is that the term ‘correctable’ suggests that the modified phenotype resulting from developmental plasticity is actually sub-optimal (i.e., needs to be ‘corrected’). In fact, the modified phenotype emerging from (enabled by) developmental plasticity might, of course, actually be advantageous under current environmental conditions – e.g., Toth and Hettyey (2018); Bautista and Burggren (2019), and Mendez-Sanchez and Burggren (2019). Not being able to resolve this issue here, this essay will nonetheless use the commonly acceptable term reversible with a parenthetical inclusion of correctable to remind the reader of the complexity of developmental plasticity.

### We Find What We Look For

Importantly, the prevailing view that phenotypic switching during critical windows of development is irreversible, likely emerges from a truth buried within developmental dogma. In fact, many of the described examples of phenotypic switching induced during an often-narrow critical window for a trait indeed appear to be irreversible (Burggren and Reyna, 2011; Daskalakis et al., 2013; Burggren and Mueller, 2015; Senner et al., 2015; Lloyd and Saglani, 2017). Moreover, stark examples such as the production of 2-headed frogs induced by hypoxia experienced during a critical window (Stockard, 1921) certainly contribute to the notion that switched phenotypes cannot be reversed. Yet, developmental critical windows are typically quite brief periods during a much longer period of overall development, and these extended developmental periods can potentially allow sufficient time for the animal to regain some or all of its normal phenotype.

As Utz et al. (2014) comment, “*Most theoretical investigations on plasticity are restricted to irreversible plasticity. i.e., the expression of a specific phenotype is determined during development and remains unchanged during the whole life of an organism*…”. As these authors go on to indicate, there are three categories of traits – non-plastic, irreversibly plastic, and reversibly plastic. An important point that Utz et al. (2014) make, and that I wish to underscore in this essay, is that most studies have focused on irreversible traits. Such traits are certainly in abundance, but do not represent the full picture of developmental phenotypic plasticity and phenotype switching. Consequently, a well populated database has been developed that is skewed toward non-reversible traits. Put differently, if one looks for irreversible phenotypic switching, one will most certainly find it. As a result, an overly restricted working definition of developmental phenotypic plasticity seems to have emerged in which the concept of reversibility has little or no traction. Against this backdrop, Lande (2014) advocates moving beyond previous theory that assumes a constant adult phenotype results from plasticity during development, to a more nuanced view that considers multiple factors. These include the cost of plasticity, the environmental variance and predictability, and developmental rates and characteristics (Lande, 2014).

**FIGURE 1:**
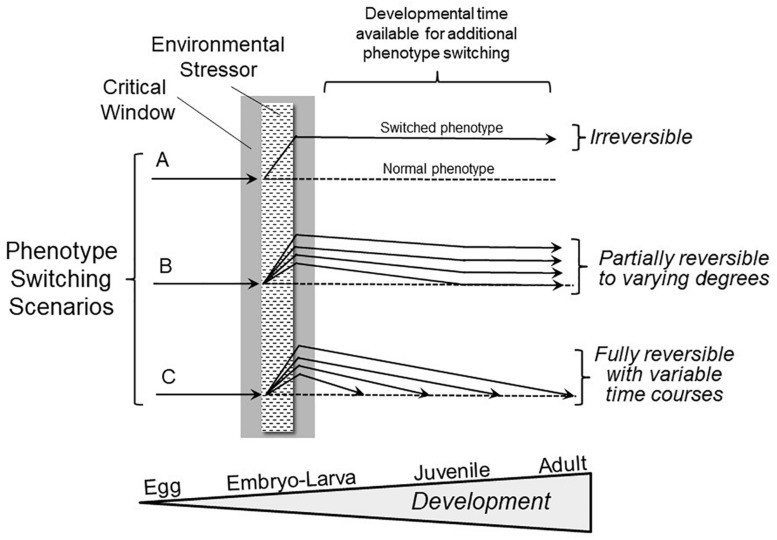
Hypothetical outcomes involving phenotype switching scenarios following exposure to adverse environments during critical windows in development. Scenario A shows the conventional, highly discussed condition of irreversible switching of phenotype induced by an environmental stressor during the critical window. Scenario B depicts a far less frequently studied situation of phenotypic switching initiated during a critical window that, in fact, is reversible to varying degrees. Scenario C indicates that there may be complete reversibility of phenotypic modifications occurring at varying rates during subsequent development. The extent and rate of phenotype reversal may relate to the extent of the difference between the altered and normal (typical) juvenile/adult phenotypes. Importantly, either a single trait might show different degrees of reversibility, or multiple traits could show a range of degrees of reversal to the normal phenotype.

Notably, an overly narrow definition has prevailed to date largely because we are not conducting experiments specifically designed to reveal reversible phenotype switching during development. Consider Hempel’s and Popper’s “Ravens Paradox,” where finding a single white raven falsifies the hypothesis that all ravens are black (Hempel, 1945; Popper, 1959). Following their logic, we only need to find one example of a phenotypic modification occurring during a developmental critical window that can be subsequently reversed (“the white raven”) to falsify the hypothesis that all phenotypic switching during development is irreversible (“all ravens are black”). If reversible phenotypic switching initially induced during the critical window were to occur, what would it actually look like? Figure 1 presents a broad view of the expression of developmental plasticity that includes reversible phenotypic switching. In the most highly studied scenario (Figure 1A) changes evoked by an environmental stressor are deemed irreversible, and as indicated earlier, there are certainly many examples of this phenomenon. However, Scenarios B and C provide for varying degrees and rates of reversibility of phenotypes initially produced as a result of stressors during the critical window.

It is important to emphasize that there are no theoretical or practical objections to reversal of phenotypic switching originating during a developmental critical windows. But, if the field of developmental biology chooses to define that particular subset of clearly non-reversible phenotypic changes occurring during development as, in fact, representing all ‘developmental phenotypic plasticity,’ we exclude real phenomena that don’t fit into that overly restrictive definition. Moreover, it means we would have to come up with yet another term to cover those examples of reversible (correctable) phenotypic switching that are induced during the critical window for development. Perhaps more efficient is simply convincing the reader that there are, indeed, clear examples of reversible phenotypic switching resulting from developmental phenotypic plasticity. Let us now consider the considerable body of evidence for reversible phenotypic plasticity.

## Reversible Developmental Phenotypic Switching: The Evidence

### Reversible (Correctable) Phenotypic Switching During Development

In the most common pattern of reversible phenotypic switching, a developing organism takes a novel developmental trajectory in response to an environmental stressor, producing a modified phenotype which may be either advantageous or deleterious. Then, when the stressor inducing this modified phenotype disappears, the organism takes a second novel developmental trajectory returning it to the pathway leading to the normal range of adult phenotypes that may well be more appropriate for the typical environmental condition (Figures 1B,C). Consider the following specific examples.

#### Invertebrates

Cold tolerance as adults can be acquired during cold exposure during early larval development in the fruit fly, *Drosophila melanogaster*. However, this modified, adaptive phenotype can be reversed during acclimation in the adult fly (Slotsbo et al., 2016). Interestingly, heat tolerance acquired during high temperature exposure as a fly larva is only partially reversible. This is reminiscent of Scenario B in Figure 1, where there can even be different degrees of reversibility for different phenotypic traits.

In the butterfly *Bicyclus anynana*, development temperature affects the size of the egg ultimately laid by the mature adults, with butterflies reared in cool temperatures producing larger eggs (Fischer et al., 2003). However, when the fully matured adults are switched to warmer temperatures for ∼10 days, they will revert to the phenotype that lays smaller eggs, as if they had been reared as larvae in cooler temperatures. Thus, these temperature-induced phenotype modifications originating in larval development are quite reversible (Brakefield et al., 2007).

Developmental phenotypic switching of mouth formation has been examined in the model nematode *Pristionchus pacificus* (Werner et al., 2017). Different culture conditions (e.g., liquid vs. agar) during development “toggle” one of two distinctive differences in mouth form phenotype. Importantly, these effects are both immediate as well as reversible when culture medium is switched, indicating that the developmental trajectory of mouth formation can be adjusted by each set of gene-environment interactions resulting from culture conditions.

#### Vertebrates

Larval newts (*Lissotriton boscai*) at stage 45–47 change pigmentation as they develop in response to different ambient backgrounds, with light, high-reflecting environments inducing depigmentation and dark, low-reflecting environments resulting in enhanced pigmentation. Yet, pigment induction is completely reversible in adults depending upon backgrounds (Polo-Cavia and Gomez-Mestre, 2017).

Temperature-metabolism relations in the turtle *Trachemys scripta elegans* are strongly affected by temperature regimes during egg incubation (Ligon et al., 2012). However, the metabolic compensation to temperature, evident in hatchlings through both measurements of resting metabolic rate and growth rate, can be later reversed irrespective of life stage and irrespective of the embryonic developmental stage at which temperature stimuli were delivered.

Nutrient restriction during the first week of development in larval *Xenopus laevis* results in the failure of neural progenitors to proliferate, but this effect can be corrected by a return to normal nutrition, affected by a subsequent 10-fold increase in cell proliferation stimulated by feeding (McKeown et al., 2017). Feeding also rescues nutrition restricted-induced decreases body length and brain tectal volume. Interestingly, the ability to rescue earlier developmental defects in the central nervous system induced by nutrient restriction during the CNS critical window (up to 7 days of development), does itself, have a critical window (≤9 days).

Numerous examples exist of reversal of modified phenotypes at the cellular and molecular levels in vertebrates. Branching morphogenesis in the renal ureteric bud/collecting duct involve new branches sprouting from the tips of existing branches rather than from the stalks of the branches. However, when the tips are removed, the stalks can form new tips, indicating that the developmental transition from tip to stalk morphology during branching is reversible (Sweeney et al., 2008). Vascular smooth muscle cells show both extensive phenotypic plasticity and diversity during development. Environmental cues (e.g., mechanical stress, oxygen partial pressure) and the genes that respond to them result in phenotypic switching of smooth muscle cells when experiencing vascular injury, for example. This phenotype switching of both form and function during development is, however, fully reversible, likely involving epigenetic markers (Owens, 2007).

#### Humans

**FIGURE 2:**
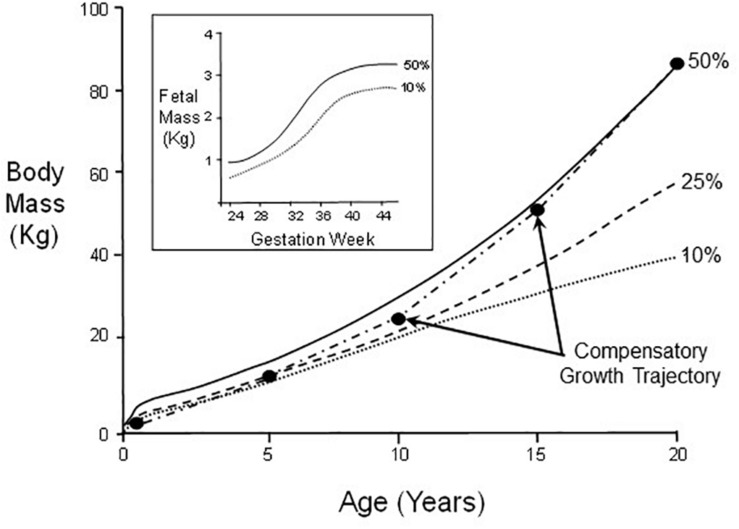
Body mass changes in male humans between birth and age 20. Indicated are growth curves for 50th, 25th, and 10th percentiles of the population. A hypothetical example of catch-up growth is also indicated. Inset: Fetal mass as a function of gestation week for 50th and 10th percentiles.

A well-documented and readily understood example of the effects of an adverse environment during critical windows for development is so-called ‘catch-up’ or compensatory growth in infants and children. Though these terms are frequently used interchangeably, a stricter definitional approach in the non-clinical literature uses “compensatory” to apply to an acceleration of growth rate, while “catch-up” more broadly allows for a return to conspecific control levels, which can be achieved by an extended period of growth with actual growth rate remaining at optimal levels (Arendt, 1997; Hector and Nakagawa, 2012). In humans, uterine nutritional insufficiency (an environmental stressor for the fetus) can result in abnormally low birth weight. However, some low birth weight infants can exhibit compensatory growth, a post-natal period of greater than typical calorie acquisition and conversion to body mass at higher rates than a normally growing infant/child (Figure 2). Compensatory growth results in an atypical developmental trajectory that ultimately restores body mass to levels typical of its normal control cohort after a few years of early childhood (Cho and Suh, 2016; Martin et al., 2017). This is not “just” slowed growth, but rather an actual departure from normal range of phenotype for the infant’s age, which is reversed (corrected) in later development.

Noteworthy is that while normal body mass *per se* is restored by adulthood (Scenario C in Figure 1), other general pathological phenotypic modifications resulting from nutrition restriction during critical windows for fetal development or early neonatal growth may not themselves be fully reversed (Scenario B in Figure 1). In the case of very low birth weight infants, this nutritional restriction during early development can lead to so-called “metabolic syndrome” in some adults, whereby increased risk exists for heart disease, stroke and type 2 diabetes (Martin et al., 2017). This underscores that any general phenotypic switching during development may simultaneously consist of both reversible *and* irreversible/pathological components (Figure 1). Consequently, experimenters need to be watchful for both categories of phenomena in experiments on developmental phenotypic switching, especially as there may be irreversible sub-components of a phenotype that are not revealed until development is complete.

Compensatory growth, and the related ‘catch-up growth’ during development, are not strictly a human or even mammalian phenomena. An extensive (but not exhaustive) meta-analysis identified these phenomena in 38 mammalian, 91 piscine, 39 avian and 30 arthropod species (Hector and Nakagawa, 2012). While not analyzed in this study, compensatory growth also occurs in amphibians and reptiles – e.g., Roark et al. (2009) and Burraco et al. (2019). Interestingly, the evolutionarily highly conserved nature of compensatory or catch-up growth suggests a high degree of biological significance. Yet, also important to note is that restoration of ‘body mass’ does not reflect appropriate restoration of all tissue types in proportion. In humans, the compensatory growth is often due to postnatal fat accumulation at the expense of muscle, in addition to the metabolic syndrome mentioned earlier (Okada et al., 2015). Thus, while body mass compensation may be obtained, the phenotype aberration caused by early undernutrition is not really fully corrected/reversed. Somewhat similarly, in lizards, regrowth of automized tails yields a structure with higher fat content than the original (Russell et al., 2015).

### “Truly Reversible” Phenotypic Switching During and After Development

More extreme cases of true reversal involve an animal actually backtracking along the normal developmental trajectory. In the hydrozoans *Turritopsis dohrnii* (the ‘immortal jellyfish’) and *Hydractinia carnea*, for example, individuals follow one of several environment-dependent alternative developmental trajectories toward the adult medusa. Astonishingly, they can actually undergo an environmentally induced regression from the sexually mature medusa back into the polyp form when faced with stress in the form of starvation or elevated temperature or salinity. This regression can occur in as little as 3 days, increasing chances of survival in the face of environmental stress (Bavestrello et al., 2000; Schmich et al., 2007; Lisenkova et al., 2017).

Similar examples of such ‘reversible acclimation’ have been observed in the acorn barnacle, *Balanus glandula*. When adult barnacles reared in quiet water are transferred to elevated seawater flows, they can regain larval segments of their feeding legs lost during typical development (Kaji and Palmer, 2017).

Physiological reversal along developmental pathways has been documented in larvae and adults of the American bullfrog, *Lithobates catesbeianus* (Santin and Hartzler, 2016). Neuroventilatory responses to CO_2_ and O_2_ involved in modulating lung breathing were reduced in aquatic overwintering adult bullfrogs. More than just attenuated, however, the gas sensitivity profiles actually reverted to that of water larvae tadpoles.

Mature human osteoblasts subjected to simulated microgravity show altered pro-osteogenic determinants and a downregulation of bone differentiation marker and adhesive proteins. In a complete reversal of the normal developmental process, this pattern of cellular dedifferentiation allows reacquisition of migration potential by the primary osteoblasts (Gioia et al., 2018).

**FIGURE 3:**
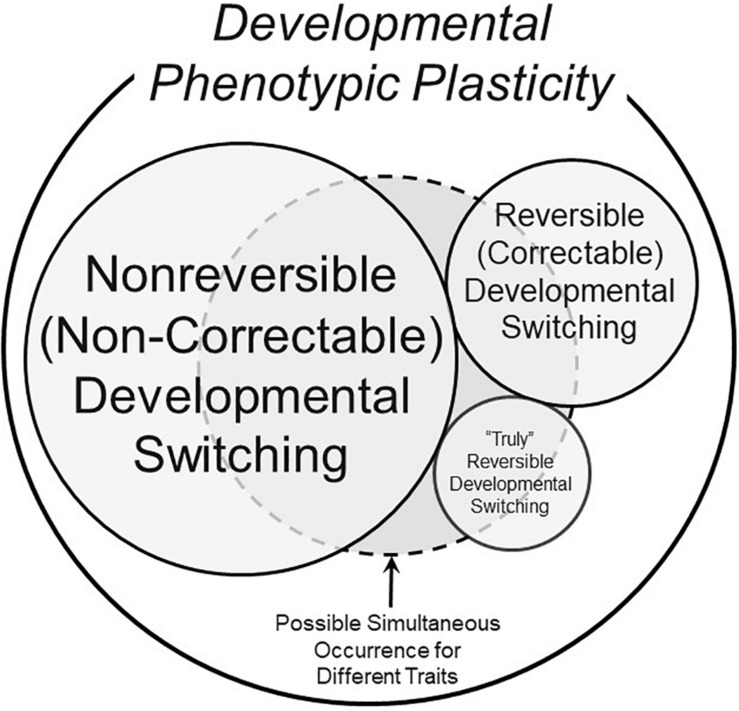
A schematic of possible components of the overall phenomenon of developmental phenotypic plasticity. This Venn diagram makes the assumption that not all phenotypic changes induced by stressors during developmentally critical windows are non-reversible (non-correctable) – see text for further discussion of definitions. The different sizes of the circles and text are meant to convey the very approximate prevalence of the form of developmental switching. The dashed circle represents the fact that all three types of developmental phenotypic switching could occur simultaneously in an animal, each for a different trait.

The examples of truly reversible development above bring into stark relief the many exceptions to the common viewpoint that modified phenotypes with their origins within developmental critical windows can, indeed, be reversible. Thus, a more comprehensive view of phenotypic switching enabled by developmental phenotypic plasticity is presented in Figure 3, which includes developmental phenotypic switching in three categories: traditional non-reversible (non-correctable), reversible (correctable) and “truly” reversible.

### Non-exclusivity of Types of Phenotype Switching

Not all traits affected by development in a single animal necessarily follow the same pattern. As Lande (2014) indicates, “…*many phenotypic characters of an individual develop and fluctuate continuously and reversibly throughout life in response to environmental change.”* Important to note is that, all too often (at least to this animal physiologist), “phenotype” refers to easily observed morphological features (e.g., size, color). In fact, an organism could for example be switching its physiology, biochemistry and behavior but not its gross external physiology. There may also be complex, subtle differences between developing phenotypic traits in terms of rates of change and metabolic associated costs (Lande, 2019). Likely most animals undergoing phenotypic switching in heterogeneous environments are ‘mosaics’, with some characters changing and others not, depending on the environmental cues.

Yet, as also emphasized above, we know that some characters do appear to become fixed during developmental critical windows. None of these scenarios are mutually exclusive. The domain circumscribed by the dashed line in Figure 3 indicates how, within a single animal, some traits could be non-reversible (non-correctable), others could be quite reversible (correctable), and still other traits might actually regress as an example of true reversibility.

As a conceptual example of multiple patterns of plasticity and subsequent phenotype switching within a single individual, consider an animal who’s pulmonary morphometrics may be permanently altered during development because of hypoxic exposure during a critical window for lung development. However, the rate and depth at which they are ventilated at rest in the adult (physiology) could be a highly plastic physiological regulatory trait. As another example, a fish’s musculature for swimming (morphology) could become fixed by experiences during development, but the characteristics of that fish’s basic schooling (behavior) as an adult could be reset by changes in fish density – thus creating the phenotype switching ‘mosaic’ referred to above. Unfortunately, empirical evidence of such changes is scarce. We know that bullfrogs exposed to hypoxia at various stages in larval development and as adults show highly differential responses associated with the timing of the hypoxic exposure. Larvae show highly plastic morphological characters associated with gas exchange, but these characters become fixed in the adult and are no longer affected by hypoxia (Burggren and Mwalukoma, 1983). In contrast, the same hypoxic exposures cause little change in the red blood cell properties, including blood P_50_, of larvae, whereas these variables remain high plastic in the adults (Pinder and Burggren, 1983). In the quail (*Coturnix coturnix*) and the chicken (*Gallus domesticus*), hypoxia experienced during different periods of embryonic incubation induces phenotypic switching of some morphological, physiological and hematological characters, but not others (Dzialowski et al., 2002; Chan and Burggren, 2005; Burggren and Elmonoufy, 2017). Certainly, more experiments designed to reveal specific patterns of phenotypic switching are warranted. To reiterate a point made earlier, if we start looking for these various forms of developmental phenotypic switching, we are likely to find them.

## Reversible Phenotype Switching as a Bridge During Periods of Unpredictable Climate Change

Having made a case for reversible (correctable) phenotype switching enabled by developmental phenotypic plasticity, what might we speculate to be the selection pressures leading to its evolution? Advantages to *mature* organisms of reversible phenotype switching, as well as its evolution, have received considerable attention – for an entry into the literature see Gabriel (2005), Gabriel (2006), Piersma and van Gils (2010), Berman (2016), Chen et al. (2016), Pfab et al. (2016), Lazaro et al. (2019), and Ratikainen and Kokko (2019). Yet, as Beaman et al. (2016) state, “…*the evolution of reversible acclimation can no longer be viewed as independent from developmental processes.*” Indeed, the phenomenon of ‘developmental bias’ is increasingly becoming an important component of understanding evolutionary process (Uller et al., 2018; Parsons et al., 2019). Unfortunately, relatively few studies have explicitly collected experimental data to determine the adaptive advantages of reversible phenotype switching in developing, as opposed to mature, organisms. Such data, if available, could then be used to infer and model the evolution of reversible (correctable) plasticity. Yet, we can still ask the question “*Why might such reversibility be important*”?

Many common environmental stressors are short-term, variable in magnitude and non-predictable in occurrence – e.g., Burggren (2018, 2019). Thus, the development of alternative traits may be adaptive for one set of environmental conditions, but maladaptive for another set which may quickly follow, especially if either the switched phenotype of the environmental conditions are extreme (Chevin et al., 2010; Chevin and Hoffmann, 2017; Burggren, 2018; Pelster and Burggren, 2018). For example, consider the development of the gills in a developing freshwater fish that typically dwells in air saturated water. The ability to develop enlarged branchial surfaces could be life-saving should temporary aquatic hypoxia occur. However, once the environment returns to its normal condition of air saturation, retaining an enlarged gas exchange surface into adulthood, or even into just the next developmental stage, is likely to be metabolically very costly. This additional cost arises from maintaining osmoregulatory balance that is challenged by the inward flood of water across the larger gill surface area. The ability to ‘shed’ a trait or suite of traits by reversing (correcting) a switched phenotype arising during development is likely to be highly adaptive to the developing animal or its adult form. In contrast, fixed phenotypes at the end of the developmental period are more likely to result in phenotype-environment mis-matches (Gomulkiewicz and Kirkpatrick, 1992; Padilla and Adolph, 1996; Dukas, 1998; Snell-Rood, 2012). Thus, one could anticipate a strong selection pressure for a form of plasticity that allowed reversible phenotype switching in developing organisms, especially when environmental stressors cycle at shorter frequencies than the life span of the organism (Gabriel, 2005, 2006). Mathematical modeling has shown that reversible phenotype switching can be beneficial, even in rhythmically and slowly changing environments (Pfab et al., 2016).

**FIGURE 4:**
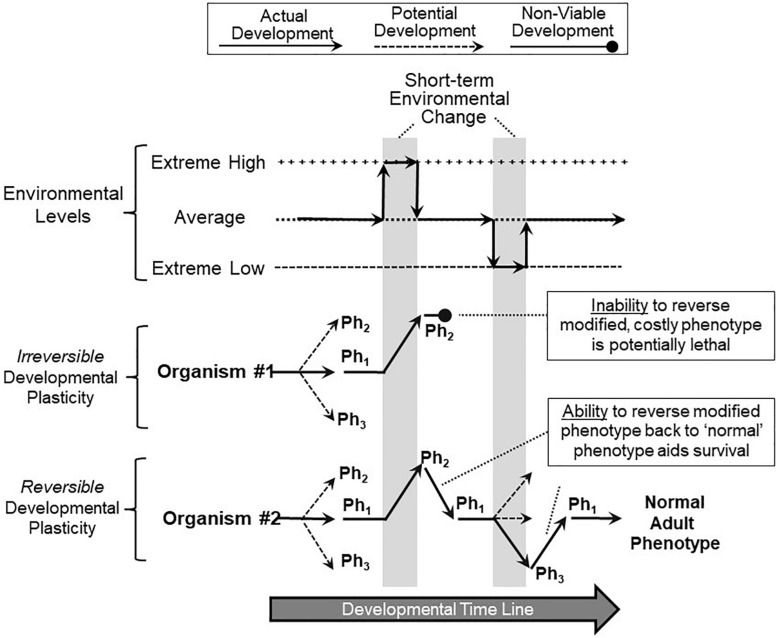
Implications of reversible and non-reversible developmental phenotypic plasticity. In this scenario, two organisms are tracked during their development as they experience multiple, short-term extreme environmental changes (vertical gray bars). Both Organism #1 (red) and Organism #2 (blue) respond to the first extreme environmental change by switching from their normal range of phenotypes (Ph_1_) to a specialized, adaptive phenotype (Ph_2_) that allows continued survival in that environment. However, Organism #1, lacking the ability to reverse (correct) its phenotype that was suitable for the extreme high environment, is now non-viable when the environment reverts back to more typical conditions as development continues. Organism #2, which similarly responded to the first environmental change with a switched phenotype (Ph_2_), has the ability to reverse or correct the costly or even lethal modified phenotype back to normal ranges (Ph_1_) when the environment returns to normal, aiding its survival. Organism #2 can also move to other phenotypes (Ph_3_) and revert back to normal ranges (Ph_1_) as subsequent environmental swings occur. Thus, in this overall scenario, reversible phenotypic switching during development heavily favors survival of the developing organism.

Reversibility of developmental phenotypic switching may be an important mechanism of surviving climate change. Simply put, the ability to reverse short-term phenotypic changes during development may be key to a species surviving short-term climate changes, especially when extreme and stochastic. Figure 4 shows a hypothetical scenario of two different organisms, both exhibiting developmental phenotypic plasticity, and both switching phenotype during development in response to the appearance of an adverse environment and its accompanying stressors. In this particular scenario, the short-term adverse environment then quickly and briefly returns to ‘normal’ (average) before actually changing to an adverse condition in the other direction. This might resemble extreme short-term weather events – for example, temperature swings – but could also mimic changes such as salinity or water availability. Let us further assume that the switched phenotype, essential for survival in a newly extreme environment, turns out to be costly, perhaps even lethal, for that organism when returned to a normally experienced or especially to a extreme low environment (as in the example of increased fish gill area, above). Under these circumstances, Organism #1, which is unable to reverse a switched phenotype, becomes non-viable and may even fail to develop (Figure 4). In contrast, Organism #2, capable of reversible phenotype switching, can revert to the original phenotype, continue development and live on to reproduce (Figure 4). Of course, there is a cost to all forms of plasticity (Snell-Rood, 2012; Lande, 2014; Pelster and Burggren, 2018), and not all phenotype switching is adaptive, especially in a stochastic environment (Padilla and Adolph, 1996; Burggren, 2018).

## Costs and Benefits of Reversible Phenotypic Switching

Having indicated that reversible phenotypic switching during development can indeed occur, a key question emerges: *How costly is reversible phenotypic switching between different developmental pathways or trajectories?* The answer hinges on the energetic/metabolic cost of developmental phenotypic switching between a modified phenotype and the normal range of phenotypes – and back. In a meta-analysis of phenotypic switching in 23 plant and animal species, the fitness costs of developmental plasticity were often found to be quite low (Van Buskirk and Steiner, 2009). Metabolic costs in particular of any magnitude can manifest themselves in terms of elevated metabolic levels or in the form of slowed growth. In fishes, physiological phenotype switching and reversal, occurring with acclimatization or laboratory acclimation, incurs variable degrees of metabolic costs (Jayasundara and Somero, 2013; Esbaugh et al., 2016). Phenotypic switching of behavioral phenotypes can be metabolically expensive given the high energetic cost of neural function and the additional neurons and neural connections that may be required (Hampson, 1991; Sporns et al., 2000; Snell-Rood, 2012; Karbowski, 2019). Changing morphological characters during development also has its costs, of course. For example, adaptive changes in head size in developing Australian tiger snakes (*Notechis scutatus*) resulted in slower rates of growth (Aubret and Shine, 2010). Morphological phenotype switching induced by predator cues in the developing freshwater crustacean *Daphnia pulex* had little apparent direct cost, but these may have been masked by an overall reduction in metabolic rate (Scheiner and Berrigan, 1998).

While in some instances the cost of a phenotypic switch and its reversal may not be prohibitively high, multiple cycles of switching caused by an environmental stressor followed by a reversal back to the normal range of phenotypes may be detrimental. Energetic reserves may be reduced or even exhausted, associated with oxidative stress being incurred. Developmental time and trajectories or even longevity even affected (Snell-Rood, 2012), to mention just a few negative consequences. Of course, the cost may pivot on the ability to acquire resources before, during and after each phenotype switching cycle.

## Conclusion

Consideration of climate change in a developmental context becomes all the more useful when expanding the concept of developmental phenotypic switching to include switching that is reversible subsequent to the critical window. Appreciating the potentially reversible (correctable) nature of at least some traits evoked by abrupt, bidirectional environmental changes could prove to be key to understanding and predicting how individuals, populations and species will cope during climate change, especially when the changes are highly variable and unpredictable.

## Author Contributions

WB wrote this manuscript and constructed all figures.

## Conflict of Interest

The author declares that the research was conducted in the absence of any commercial or financial relationships that could be construed as a potential conflict of interest.

## Footnotes

1 The philosopher Popper (1970) suggested that “…*we should altogether avoid, like the plague, discussing the meaning of words*.” While one can be sympathetic to his viewpoint, due diligence requires acknowledging differing definitions in the long-standing debate as to the reversibility of effects resulting from developmental plasticity. Underscoring this point, the two reviewers of this manuscript had differing opinions on whether the field viewed phenotypic switching as reversible or irreversible when it occurred during development. Ultimately, perhaps Alfred Korzybski (1879–1950), the father of the field of General Semantics, got it right when he posited that “*Whatever you say it is, it isn’t*” (Korzybski, 1933).
